# Long-term storage modifies the microRNA expression profile of cryopreserved human semen

**DOI:** 10.17305/bb.2023.9421

**Published:** 2024-02-01

**Authors:** Chuan Huang, Xi-Ren Ji, Zeng-Hui Huang, Qian Liu, Rui-Jun Wang, Li-Qing Fan, Hui-Lan Wu, Hao Bo, Wen-Bing Zhu

**Affiliations:** 1 Clinical Research Center for Reproduction and Genetics in Hunan Province, Reproductive and Genetic Hospital of CITIC-Xiangya, Changsha, Hunan, China; 2 Institute of Reproductive and Stem Cell Engineering, Basic Medicine College, Central South University, Changsha, Hunan, China

**Keywords:** Human semen, sperm cryopreservation, storage time, microRNA (miRNA) expression, miR-Let-7

## Abstract

The global practice of cryopreservation of human semen is commonplace in Assisted Reproductive Technology (ART) labs and sperm banks. However, information on the effects of long-term cryopreservation on semen is limited to clinical data summaries and descriptions. For this study, we prepared 4 semen specimens of fresh semen, 4 specimens cryostored for at least 1 year, 3 specimens cryostored for at least 5 years, 4 specimens cryostored for at least 10 years, and 3 specimens cryostored for at least 15 years. Total RNA was extracted from each sample, amplified, labeled, and mapped to the known primary microRNA (miRNA) in the miRBase database, enabling the prediction of novel miRNAs. We found that cryopreservation can lead to changes in miRNA expression, and with the increase in storage time, these changes became more pronounced. Meanwhile, the expression of let-7d-3p, let-7c-5p, and let-7i-3p miRNAs changed dynamically over cryostorage time in frozen-thawed human sperm. Furthermore, we analyzed the time-dependent dynamics of cryostorage-expressed miRNAs and their target mRNAs and found that half of the target genes were expressed in oocytes. These intersection genes were mainly enriched in cancer and cytoskeletal signaling pathways. Our findings showed that the miRNA expression profile of cryopreserved human semen is modified by long-term storage. Furthermore, as the storage time increases, the impact on human sperm becomes more pronounced in terms of miRNAs, which may have an effect on subsequent fertilization and embryonic development.

## Introduction

The first documented instance of semen cryopreservation occurred over 200 years ago [1]. The identification of glycerol as a cryoprotectant [2] and the understanding of gas liquefaction [3] have led to the development of technology that can be used to freeze and store semen at very low temperatures. Human sperm cryopreservation has become an accepted procedure in human sperm banks and Assisted Reproductive Technology labs around the world. However, cryopreservation protocols can significantly alter human sperm motility and cause intracellular and extracellular damage, including changes in sperm membrane and acrosome integrity, DNA fragmentation, and increased levels of reactive oxygen species [4]. Cryopreservation of human semen also has an impact on the sperm cytoskeleton, which changes over time [5].

Sperm cryopreservation is currently the only way to efficiently preserve male fertility. Nowadays, fertility preservation has become increasingly important for improving the quality of life of patients who have completely recovered from cancer. However, the use of frozen sperm in young cancer survivors may involve a long period time. Therefore, it is important to elucidate the potential effects of long-term cryopreservation of sperm. Although few studies report that long-term cryostorage of semen in patients with cancer [6] and qualified sperm donors in human sperm banks does not affect clinical outcomes [7], these studies have only considered pregnancy outcomes. The effects of long-term cryostorage in liquid nitrogen on sperm are not well understood due to a lack of experimental data. However, this information is essential to determine the safety of this practice.

Sperm, specialized and differentiated cells, are primarily responsible for the transmission of paternal genetic information and coding and non-coding RNAs to the oocyte [8]. These RNAs include messenger RNAs (mRNAs), ribosomal RNAs (rRNAs), and small RNAs (sRNAs), which mainly result from spermatogenesis [9, 10]. MicroRNAs (miRNAs) are small endogenous RNAs that decrease the expression of target mRNAs that contain sequences complementary to the miRNA [11]. Significant dysregulation in miRNA expression has been observed in various types of reproductive problems [12]. Furthermore, miRNAs could be involved in sperm cryo-damage [13, 14]. Thus, miRNAs could serve as critical regulators of sperm cryo-damage and cryo-resistance.

To our knowledge, documentation of the effects of long-term cryopreservation on semen is limited to clinical data summaries and descriptions. To date, there have been no reports on the use of next-generation sequencing to investigate the long-term effects of cryopreserved semen. This study aimed to analyze the impact of the duration of cryostorage on the miRNA expression in cryopreserved human semen. To compare the miRNA expression profiles of freeze-thawed semen, we collected samples that had been stored in liquid nitrogen for periods of 1, 5, 10, and 15 years. Non-cryopreserved human semen was also studied as a control.

## Materials and methods

### Study population and participants

From 1 January 2001 to 31 December 2021, the specific study subjects in this study were 18 adult males aged between 22 and 28 years who had been qualified sperm donors at the Hunan Province Human Sperm Bank of China. Demographic data, such as age, height, weight, and abstinence periods were collected. Meanwhile, clinical information, such as semen statistics before and after freezing, were also analyzed in this project.

### Study design

To minimize patient-specific variations in miRNA expression, semen specimens were randomly divided into different groups. Four samples of freshly collected semen were designated to the “0 years” group. The 0 years group was provided with four samples of freshly collected semen. Additionally, 4 samples of semen that had been cryostored for 12–18 months were collected for the “1 year” group. Three samples of semen that had been cryostored for 60–66 months were collected for the “5 years” group. Four samples of semen that had been cryostored for 120–126 months were collected for the “10 years” group. Three samples of semen that had been cryostored for 180–186 months were collected for the “15 years” group. Importantly, all samples were obtained from different donors.

### Semen analysis

Semen samples were collected by masturbation after a period of 2–7 days. Afterward, these samples were carefully kept in sterile containers [15]. As per the World Health Organization (WHO) 1999 guidelines, all specimens were then evaluated [16]. After liquefaction, the semen volume was measured by weighing within an hour of ejaculation. The sperm concentration and motility were evaluated using a Makler Counting Chamber (Sefi Medical Instruments, Haifa, Israel) and a microscope with a magnification of ×200 (Olympus BX43, Tokyo, Japan). The motility was divided into four different groups based on their different status after liquefaction: highly active (group A), lowly active (group B), non-active (group C), and immotile (group D). Briefly, the average results were documented after one replicate, in which at least 200 sperm were counted.

### Freezing and thawing

Cryostorage was used to preserve the sperm specimens in their raw form. To dilute one specimen, 1/4 volume of the freezing medium was required. Following this, the specimen was placed at room temperature for approximately 15 min to allow it to equilibrate before being stored in 1.8 mL cryotube (Thermo Fisher Scientific, USA) and cryopreservation in a controlled-rate freezer (Thermo 7451, Thermo Fisher Scientific, USA). The cryotubes were slowly cooled and moved to liquid nitrogen for cryostorage. To evaluate the sperm motility after thawing but before cryostorage, one cryotube of each specimen was thawed at 37 ^∘^C for 15 min. The standard for recruiting sperm bank donors, freezing protocol, equipment, and components of the freezing medium buffer remained constant. Nonetheless, there are many other unexpected or unrealized factors that compromise the reliability of the results.

### RNA extraction

Before total RNA extraction, each semen sample with 1 mL volume was centrifugated for 10 min at 500 *g* to remove the cryoprotectant or seminal plasma. Then the sperm pellet was resuspended in 1× PBS and centrifugated for 10 min at 500 *g* to remove PBS. Subsequently, the sperm pellet was used for RNA extraction. TRIzol Reagent (Thermo Fisher Scientific, USA) was used to extract RNA from semen, according to the manufacturer’s instructions. DNA digestion was then conducted with DNaseI (Thermo Fisher Scientific, USA). The quality of RNA was checked by assessing A260/A280 with a Nanodrop^TM^ OneC spectrophotometer (Thermo Fisher Scientific, USA). To measure the amount of qualified RNAs, Qubit3.0 with a QubitTM RNA Broad Range Assay kit (Thermo Fisher Scientific, USA) was selected.

### miRNA expression profiling

Following the manufacturer’s instructions, a KC-Digital^TM^ small RNA Library Prep Kit for Illumina^®^ (Cat: RK20307, Wuhan Seqhealth Co., Ltd., China) was used to input 3 µL (10 ng) total RNA for the preparation of miRNA libraries. A unique molecular identifier (UMI) of eight random bases was used to label pre-amplified small RNA molecules, thus avoiding duplication errors in PCR and sequencing steps. The cDNA library was separated using 6% PAGE gel. During the isolation, purification, and qualification of the bands (approximately 160 bp), Qubit3.0 was used. Finally, the sequencing of samples was completed on a Hiseq X-10 sequencer (Illumina; model PE150). Fastx_toolkit was employed to filter the unprocessed sequencing data, eliminating low-grade reads, and Cutadapt (version 1.15) was used to trim adaptor sequences. To avoid any duplication bias as a result of sequencing and preparation in library, in-house scripts were then utilized to further process clean reads. To eliminate any errors or biases from PCR amplification or sequencing, for each different sample, their de-duplicated consensus sequences were aligned to the reference human genome (ftp://ftp.ensembl.org/pub/release-75/fasta/homosapiens/) using bowtie [17] (version: 1.1.2) with default parameters. The miRDeep2 [18] (version: 2.0.0.8) package was then utilized to match the results to known primary miRNAs in the miRbase database and identify and determine the functions of novel miRNAs.

**Table 1:** Primers used for qRT-PCR validation

**miRNA**		**miRBase**	**Primer sequences (5′-3′)**
U6	Forward	NR_004394.1	CTCGCTTCGGCAGCACAT
	Reverse		AACGCTTCACGAATTTGCGT
hsa-miR-655-3p	Forward	MIMAT0003331	GGCATAATACATGGTTAACC
	Reverse		CTCAACTGGTGTCGTGGAGTC
hsa-miR-514a-3p	Forward	MIMAT0002883	GGGCCATTGACACTTCTGTG
	Reverse		CTCAACTGGTGTCGTGGAGTC
hsa-miR-506-3p	Forward	MIMAT0002878	TCGGCACCCTTCTGAGTAGA
	Reverse		CTCAACTGGTGTCGTGGAGTC
hsa-miR-143-3p	Forward	MIMAT0000435	GAAGCACTGTAGCTCCTCAACTG
	Reverse		CTCAACTGGTGTCGTGGAGTC
hsa-let-7d-3p	Forward	MIMAT0000063	GGGCCTGAGGTAGTAGGTTG
	Reverse		CTCAACTGGTGTCGTGGAGTC
hsa-let-7c-5p	Forward	MIMAT0000064	GGGCCGGTAGTAGGTTGTA
	Reverse		CTCAACTGGTGTCGTGGAGTC
hsa-let-7i-3p	Forward	MIMAT0004585	GGCCCTGCGCAAGCTACT
	Reverse		CTCAACTGGTGTCGTGGAGTC

qRT-PCR: Quantitative reverse-transcription polymerase chain reaction; miRNA: MicroRNA.

### Data analysis

All figures were generated using R software (http://www.r-project.org, version: 3.5.1), except for the miRNAs target gene enrichment analysis map, which was generated using Metascape online software [19] (https://metascape.org/gp/index.html#/main/step1) based on default parameter analysis. The ggpubr package’s ggpie function was used to create the pie chart. The pheatmap package was used to generate the sample correlation clustering heat map, with “average” as the clustering mode. The ggplot2 package was used to create violin charts, bar charts, and line charts. The specific and highly expressed miRNAs at different storage times were analyzed using the FindAllMarkers function of the Seurat package. Differential expression analysis of miRNAs between different storage time groups and fresh groups was performed using the FindMarkers function of the Seurat package, and the volcano map of the differential miRNA expression was drawn using the EnhancedVolcano package. The Venn diagram was drawn using the VennDiagram package. The storage time-dependent trend analysis of miRNA was drawn using the Mfuzz package analysis, and target gene prediction was performed using the multiMiR package. We only used data that had been verified by luciferase experiments for follow-up analysis. The data of mRNA genes expressed in oocytes were downloaded from a previously published study [20].

### Quantitative reverse-transcription polymerase chain reaction (qRT-PCR)

By randomly selecting and confirming the expression of seven miRNAs by qRT-PCR, we validated the high-throughput sequencing results. For resampling, we mixed sperm from five individual males to prepare semen samples. Primers were designed using Primer Premier 5.0 software (Table 1) and homologous counterparts from the miRBase database. Following the manufacturer’s instructions, qRT-PCR was performed on a Step One Plus real-time PCR system (Applied BioSystems, USA), using the SYBR PrimeScript miRNA RT-PCR Kit (Takara Biotech, China).

### Ethical statement

All donors who visited the human sperm bank for the first time signed consent forms, granting permission for their semen information to be used for scientific projects. The present study was approved by the Ethics Committee of the Central South University (2022-KT149).

### Statistical analysis

Variable data are presented as means ± standard deviation (SD) and divided into 5 groups based on storage periods: 0 years, 1 year, 5 years, 10 years, and 15 years. To compare variations between different groups for continuous variables, the nonparametric Kruskal–Wallis test was used, while chi-squared or Fisher’s exact tests were used to analyze categorical variables. The Statistical Package for the Social Sciences (SPSS) 19.0 was utilized to analyze the statistical data, with a *P* value of less than 0.05 deemed statistically significant.

## Results

### Participant characteristics

Table 2 provides a summary of the demographic and clinical data of the sperm donors in this study. No differences were found among the five groups in age (*P* ═ 0.83), height, weight, body mass index, abstinence times, and sperm parameters before and after freezing.

**Table 2:** Demographic and clinical information of the study participants

	**0-years storage (*N* ═ 4)**	**1-year storage (*N* ═ 3)**	**5-years storage (*N* ═ 3)**	**10-years storage (*N* ═ 4)**	**15-years storage (*N* ═ 3)**	
**Demographic variables**	**Mean (SD)**	**Mean (SD)**	**Mean (SD)**	**Mean (SD)**	**Mean (SD)**	**Difference between the five or four groups *P* value**
Age (years)	23.5 (1.7)	24.8 (2.0)	25.0 (0.8)	24.0 (0.7)	24.7 (2.1)	0.83
Height (m)	1.7 (0.02)	1.7 (0.04)	1.7 (0.07)	1.7 (0.02)	1.7 (0.01)	0.92
Weight (kg)	57.8 (2.9)	56.5 (3.8)	66.3 (5.8)	61.3 (2.5)	57.0 (6.7)	0.21
BMI (kg/m^2^)	19.5 (0.9)	19.0 (1.0)	21.7 (0.7)	21.5 (0.3)	19.0 (2.4)	0.24
Abstinence time (days)	4.5 (1.1)	5.5 (1.1)	4.7 (0.5)	5.8 (1.6)	4.7 (0.5)	0.53
Semen volume (mL)	3.2 (0.8)	3.6 (1.3)	3.6 (1.5)	3.5 (1.1)	3.4 (1.2)	0.38
Sperm concentration (million/mL)	63.1 (2.3)	63.3 (3.1)	67.4 (4.7)	65.5 (2.9)	68.1 (5.2)	0.23
Total sperm count (million)	191 (12.7)	198 (13.0)	211 (16.0)	205 (19.3)	216 (14.8)	0.41
Pre-freeze semen motility (%) (a+b)	51.0 (0.2)	52.1 (1.6)	51.8 (1.2)	53.4 (2.5)	56.4 (2.1)	0.36
Pre-freeze total motility sperm count (million)	103.3 (5.8)	101.9 (7.9)	115.9 (18.5)	119.0 (16.5)	120.5 (12.6)	0.20
Frozen volume per cryotube (mL)	/	1	1	1	1	/
Post-thaw semen motility (%) (a+b)	/	42.2 (2.2)	43.0 (2.3)	43.6 (2.2)	38.0 (1.2)	0.06
Post-thaw total motility sperm count (million)	/	20.2 (2.2)	22.2 (2.53)	22.5 (3.2)	18.0 (2.2)	0.21

SD: Standard deviation; BMI: Body mass index.

### miRNA expression characteristics after different sperm cryostorage durations

**Figure 1.**
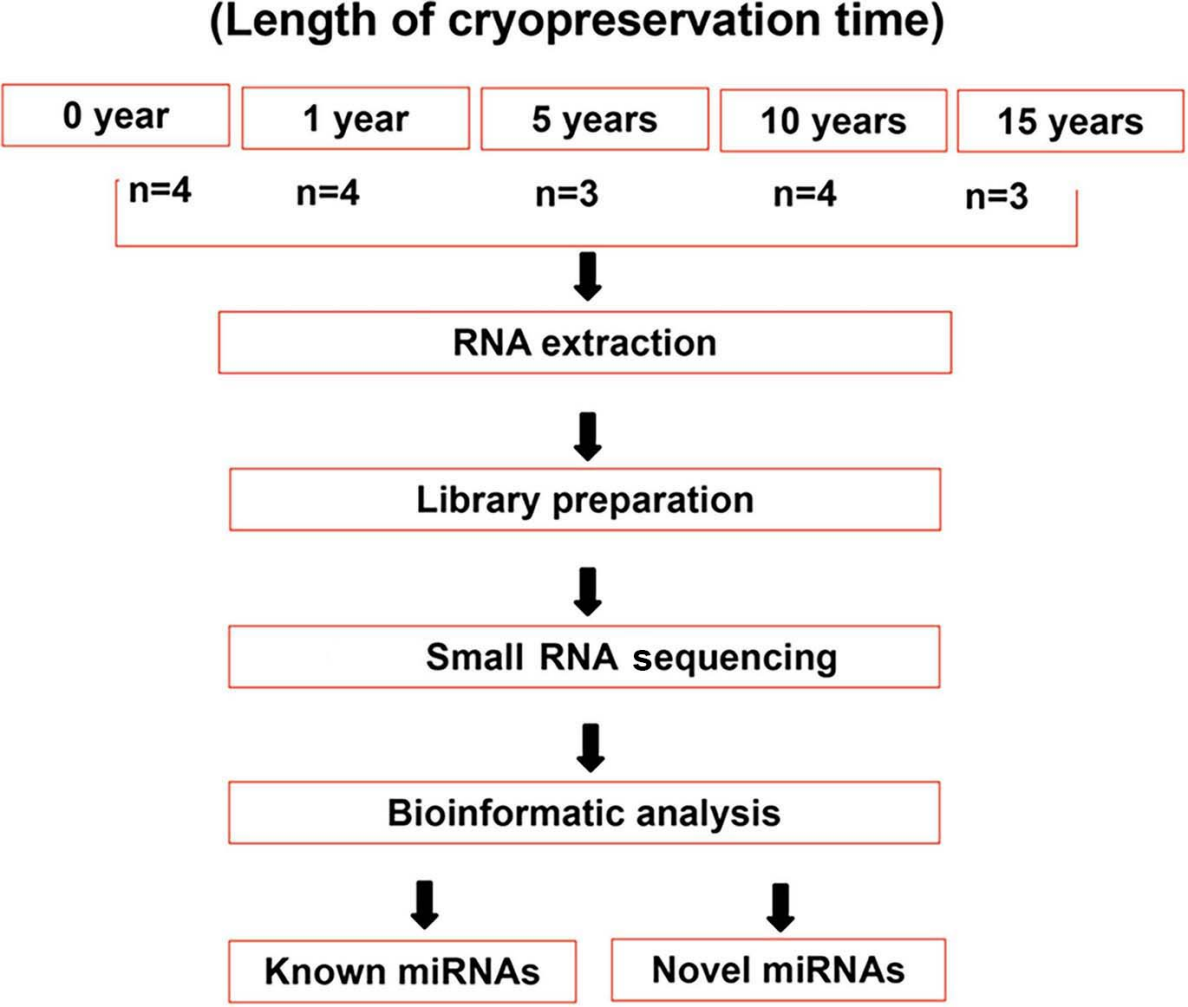
**Flowchart showing the design of the present study.** miRNA: MicroRNA.

**Figure 2.**
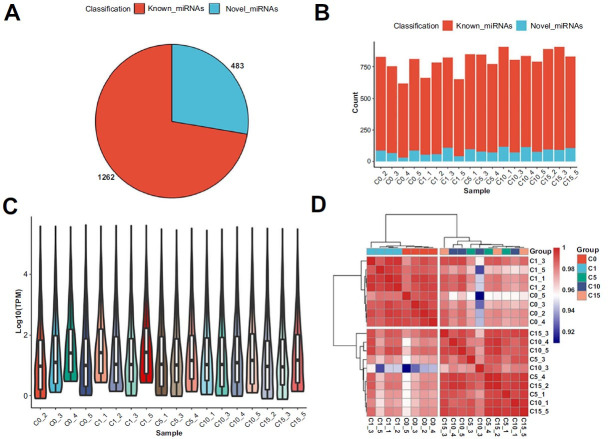
**miRNA expression.** (A) The number of known and novel miRNAs detected based on small RNA sequencing; (B) The number of known and novel miRNAs detected in each sample; (C) Violin diagrams of miRNA abundance in each sample; (D) Heatmap showing the miRNA expression correlation of each sample. miRNA: MicroRNA.

Figure 1 shows the design of the present study. Compared with the spectral library, 1745 miRNAs were identified, of which 1262 were known miRNAs and 483 were novel (Figure 2A). Approximately 650–750 known miRNAs were detected in each sample, and the number of miRNAs detected in each group was similar (Figure 2B). However, the violin plot showed that the relative abundances of miRNAs were higher in the 0 years and 1-year groups than in the other groups (Figure 2C). There was a stronger relationship between miRNA expression in the 0 years and 1-year groups, and in 5, 10, and 15 years groups (Figure 2D). These results suggest that human sperm cryopreservation for one year may have little effect on the miRNA expression profile. However, sperm cryopreservation for more than five years could significantly modify the miRNA expression profile.

### Identification of differentially expressed miRNAs at different sperm cryostorage durations

**Figure 3.**
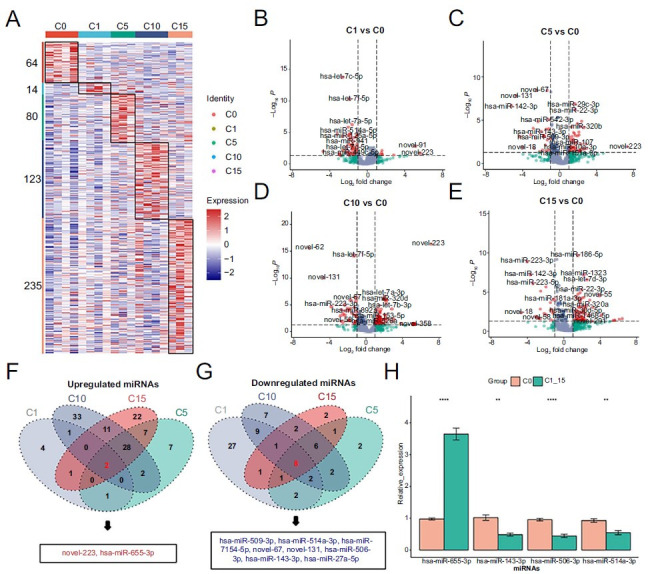
**Expression of miRNAs was significantly different in samples with different cryostorage durations.** (A) Heatmap showing the differentially expressed miRNAs in samples after different cryostorage durations; (B) Volcano map representing the miRNA log-fold changes and *P* values. Volcano plots of the differentially expressed miRNAs in the 1-year and fresh groups; (C) Volcano plots of the differentially expressed miRNAs in the 5-years and fresh groups; (D) Volcano plots of the differentially expressed miRNAs in the 10-years and fresh groups; (E) Volcano plots of the differentially expressed miRNAs in the 15-years and fresh groups; (F) Co-expression of upregulated common differentially expressed miRNAs at different cryostorage durations; (G) Co-expression of downregulated common differentially expressed miRNAs at different cryostorage durations; (H) qPCR verification of four common differentially expressed miRNAs. miRNA: MicroRNA.

The miRNAs that were differentially expressed at different cryostorage times were compared and are shown as a heat map (Figure 3A). Cryopreservation could lead to changes in miRNA expression, that became more pronounced with the increase of storage time (using a nominal *P* value cutoff of 0.05). There were 78, 144, 187, and 299 more miRNA differences in the 1, 5, 10, and 15 years groups, respectively, compared with the 0 years group. The details of the differentially expressed miRNAs are provided in Figure 3B–3E. Ten differentially expressed miRNAs were found to overlap between all cryopreservation groups compared with the fresh group (Figure 3F and 3G). Eight of these miRNAs (hsa-miR-509-3p, hsa-miR-514a-3p, hsa-miR-7154-5p, novel-67, novel-131, hsa-miR-506-3p, hsa-miR-143-3p, and hsa-miR-27a-5p) were found at higher levels in the cryopreservation groups, and two were found at lower levels (novel-223 and hsa-miR-655-3p). To validate the sequencing data, RT-qPCR was used to assess the relative expression level of four randomly selected differentially expressed miRNAs (Figure 3H). The results from RT-qPCR were in agreement with the RNA-seq data for all miRNAs, thereby demonstrating the accuracy of the RNA-seq results.

### Cryostorage time-dependent miRNA expression trends

**Figure 4.**
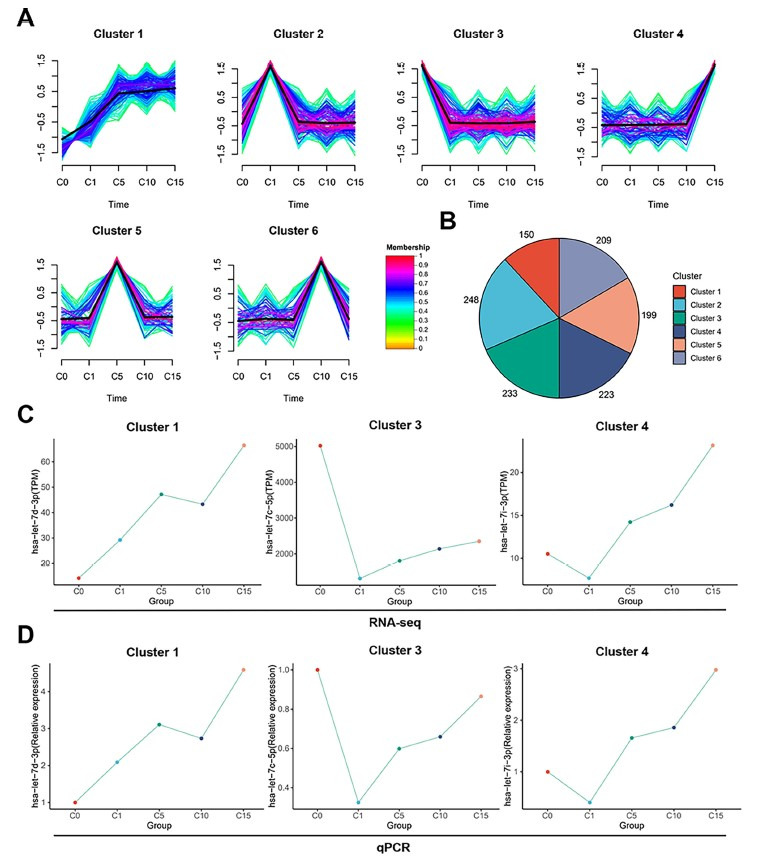
**Cryostorage time-dependent expression of miRNAs.** (A) All miRNAs could be divided into six clusters by cryostorage time-dependent miRNAs expression trend analysis; (B) Pie chart showing the number of miRNAs for each cluster; (C) Several Let-7 family miRNAs were dynamically expressed in cluster1/cluster3/cluster4; (D) qPCR verification of the dynamic expression of let-7d-3p, let-7c-5p, and let-7i-3p miRNAs. miRNA: MicroRNA.

We clustered all known miRNAs and found 6 cryostorage time-dependent trends (cluster 1 containing 150 miRNAs; cluster 2 containing 248 miRNAs; cluster 3 containing 233 miRNAs; cluster 4 containing 223 miRNAs; cluster 5 containing 199 miRNAs, and cluster 6 containing 209 miRNAs) (Figure 4A and 4B). Multiple Let-7 family members with dynamic expression were found in clusters 1, 3, and 4 (Figure 4C). The expression of these members of the Let-7 family of miRNAs (hsa-let-7d-3p, hsa-let-7c-5p, and hsa-let-7i-3p) at different storage times was evaluated by RT-qPCR in order to validate the dynamic expression pattern of the Let-7 family in clusters 1, 3, and 4. (Figure 4D). Results from RT-qPCR were consistent with the dynamic expression pattern, which indicated that the clusters 1, 3, and 4 results were reliable. Thus, the Let-7 family of miRNAs (hsa-let-7d-3p, hsa-let-7c-5p, and hsa-let-7i-3p) may be involved in the changes that occur during sperm storage.

### Target gene prediction and enrichment analysis of differentially expressed miRNAs

**Figure 5.**
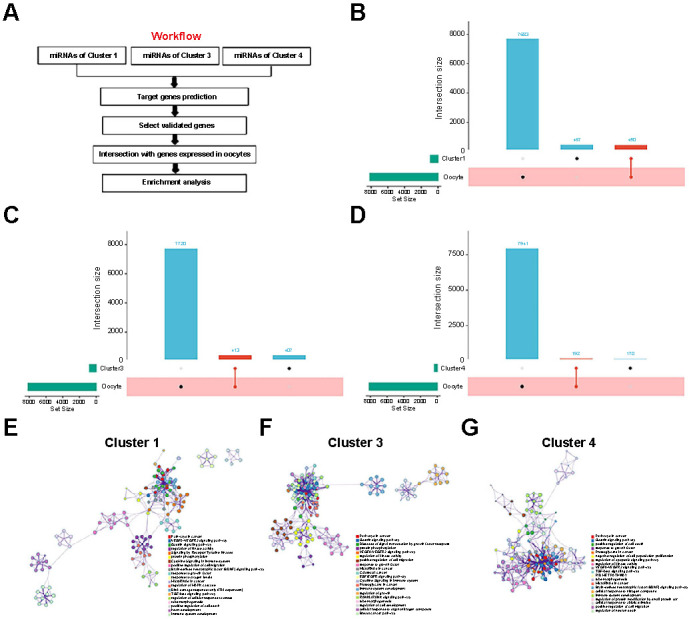
**Potential function analysis of differentially expressed miRNAs.** (A) Flowchart of miRNAs target gene enrichment analysis; (B) UpSet plots of the miRNA target genes in cluster 1 and verified genes expressed in oocytes; (C) UpSet plots of miRNA target genes in cluster 3 and verified genes expressed in oocytes; (D) UpSet plots of miRNAs target genes in cluster 4 and verified genes expressed in oocytes; (E) Network relationship plots showing intersection of miRNA target genes in cluster 3 and genes expressed in oocytes; (F) Network relationship plots showing intersection of miRNAs target genes in cluster 3 and genes expressed in oocytes; (G) Network relationship plots showing intersection of miRNAs target genes in cluster 4 and genes expressed in oocytes. miRNA: MicroRNA.

Figure 4A shows the analyses performed to choose miRNAs and their target genes. All differentially expressed miRNAs from clusters 1, 3, and 4 had predicted target genes. We extracted only predicted target mRNA genes that were been verified by luciferase experiments and compared them with mRNA genes expressed in oocytes. Half of the target genes in clusters 1, 3, and 4 were expressed in oocytes (Figure 5B–5D). Of the 917 target genes in cluster 1, 450 were expressed in oocytes. Similarly, 407 of 820 target genes in cluster 3 and 192 of 362 target genes in cluster 4 were expressed in oocytes. These intersection gene enrichment analyses are shown in Figure 5E–5G. The intersection genes were mostly distributed and associated with cancer and cytoskeletal signaling pathways.

## Discussion

Despite advances in sperm cryopreservation, the underlying biological and biochemical processes remain obscure. There are several factors that are believed to impair sperm quality during the freezing and thawing process, such as sudden temperature changes, ice formation, and osmotic stress. Furthermore, there is a lack of knowledge on the new aspects of sperm cryobiology, and no experiment data regarding the effect of storage time on sperm are currently available. Hence, we performed miRNA expression profiling of freeze-thawed semen after 1, 5, 10, and 15 years of storage in liquid nitrogen and demonstrated that the duration of storage could affect the miRNA expression profile of cryopreserved human semen. This finding could explain our recent clinical studies indicating that long-term storage negatively influenced survival rates after thawing, in terms of pregnancy and abortion rates [7].

miRNAs can inhibit translation and gene expression during key developmental transitions, such as the oocyte-to-zygote transition, zygotic genomic activation, and initial embryonic development in mammals. Cryopreservation modifies the miRNA expression profile of semen in several mammals. A previous study on bull sperm identified 55 differentially expressed miRNA fragments between frozen and fresh sperm [8]. There were also changes in the expression of 135 miRNA between frozen and fresh boar semen [21]. Further, 21 and 95 differentially expressed miRNAs were identified in frozen-thawed and fresh sperm samples from humans and mice, respectively [13]. In this study, we observed that human sperm cryopreservation for one year had little effect on the miRNA expression profile. However, sperm cryopreservation for more than 5 years could significantly modify the miRNA expression profile. Furthermore, 10 differentially expressed miRNAs were found in all the cryopreservation groups compared with the fresh group. Among these, hsa-miR-655-3p [22], hsa-miR-506-3p [23], hsa-miR-143-3p [24], and hsa-miR-27a-5p [25] could cause a decreased fertilization rate in in vitro fertilisation and intracytoplasmic sperm injection using cryopreserved sperm, as these miRNAs are all related to the cell cycle. miR-509-3p could be used as a biomarker to predict implantation success [26]. Target genes for miR-514a-3p participate in spermatogenesis and sperm functions [27]. The precise function of these miRNAs in sperm remains a mystery, yet their expression may be linked to decreased motility and augmented apoptosis in thawed human sperm, thus demonstrating their essential biological role.

Let-7 is one of the earliest known miRNAs and was first discovered in the *Caenorhabditis elegans*. It is also the first miRNA to be discovered in humans [28]. The Let-7 family consists of 13 highly conserved members with the same seed sequence, targeting multiple genes in various cell types. Let-7 family members play roles in sperm motility, fertility, early embryo development, and pregnancy. Members of the Let-7 family play a central role in inducing embryonic diapause; Let-7 overexpression had an inhibitory effect on the differentiation and implantation of human embryo surrogates, as well as increasing the longevity of human blastocysts when cultured in vitro [29]. Higher expression of let-7a, -7d, and -7e was found in sperm with a high percentage of morphological abnormalities or low motility, and these changes in expression have been associated with changes in morphology and motility [30]. Let-7 family miRNAs are likely involved in spermatogenesis and show high expression levels in type B spermatogonia cells and primary spermatocytes [31]. In our study, we found that let-7d-3p, let-7c-5p, and let-7i-3p miRNA expression changed dynamically over cryostorage time in frozen-thawed human sperm. Despite their scarce presence in early embryos, Let-7 miRNAs are the most abundant maternal miRNAs, making them quite remarkable [32]. However, the expression of Let-7 miRNA, especially let-7d-3p, increased with the duration of cryopreservation. LIN28 proteins have been found to bind Let-7 pre-miRNAs and various mRNAs, and are linked to pluripotency, development, growth, and metabolism [33]. When LIN28A was knocked down, the majority of embryos arrested between the 2- and 4-cell stages, suggesting that LIN28A plays a role in nucleogenesis during early development [34]. Our findings suggest that as the duration of sperm cryostorage increases, there’s an overexpression of Let-7 miRNAs, especially let-7d-3p. This overexpression may inhibit LIN28 expression, potentially having an adverse effect on early embryonic development.

We conducted a study to examine the differentially expressed miRNAs and their target mRNAs. Of the target genes, half were expressed in oocytes. These target genes may be involved in fertilization and embryonic development. The cancer signaling pathways, such as cAMP-PKA, PI3K/AKT, and TGF-β, are heavily implicated in the target mRNAs of this study. These pathways are essential for sperm capacitation and acrosome development [12] and are thus highly enriched. Activation of ion channels, like the CatSper Ca^2+^ channel, can bring about the signal transduction factors required for initiating the cAMP-PKA signaling pathway and the subsequent stages of sperm capacitation [35]. Ca^2+^ is an essential element for sperm capacitation, fertilization, and egg activation. Additionally, the TGF-β pathway is essential for embryonic development [36] and regulates the transcription of genes that control cell growth, differentiation, and death [37]. We, therefore, hypothesize that long-term cryostorage of human semen might abnormally induce several signaling pathways. Further research on the correlation between these signaling pathways and sperm cryopreservation is therefore important.

This study had a few limitations, including the small sample size. The results should be taken with caution as the sample size is quite limited and need further studies to support the present results, although stringent criteria for identifying differentially expressed miRNAs were used. Furthermore, despite the lack of differences in demographic characteristics between the various groups, there may be inter-individual variability as all semen samples were collected from different individuals. In addition, more research is necessary to ascertain whether long-term cryopreservation affects other molecular mechanisms.

## Conclusion

Our findings indicated that, to the best of our understanding, the miRNA expression profile of cryopreserved human semen can be altered by long-term storage. This is the first study of its kind to explore the impact of storage time on miRNA expression profiles in such semen. Furthermore, as storage time increases, the impact on human sperm miRNA expression became more pronounced and may affect subsequent fertilization and embryonic development. Consequently, sperm banks ought to supply sperm in the sequence of cryopreservation.
